# A Double-Antibody Sandwich ELISA for Sensitive and Specific Detection of Swine Fibrinogen-Like Protein 1

**DOI:** 10.3389/fimmu.2021.670626

**Published:** 2021-04-23

**Authors:** Xin Zhang, Haipeng Zhu, Xu Zheng, Yunjie Jiao, Lulu Ning, En-Min Zhou, Yang Mu

**Affiliations:** ^1^ Department of Preventive Veterinary Medicine, College of Veterinary Medicine, Northwest A&F University, Yangling, China; ^2^ Scientific Observing and Experimental Station of Veterinary Pharmacology and Diagnostic Technology, Ministry of Agriculture, Yangling, China

**Keywords:** swine FGL1, detection, monoclonal antibody, double-antibody sandwich ELISA, PRRSV-negative and -positive pig sera

## Abstract

Fibrinogen-like protein 1 (FGL1), a member of the fibrinogen family, is a specific hepatocyte mitogen. Recently, it has been reported that FGL1 is the main inhibitory ligand of lymphocyte activating gene 3 (LAG3). Furthermore, the FGL1-LAG3 pathway has a synergistic effect with programmed death 1 (PD-1)/programmed death ligand 1 (PD-L1) pathway and is regarded as a promising immunotherapeutic target. However, swine FGL1 (sFGL1) has not been characterized and its detection method is lacking. In the study, the sFGL1 gene was amplified from the liver tissue of swine and then inserted into a prokaryotic expression vector, pQE-30. The recombinant plasmid pQE30-sFGL1 was transformed into JM109 competent cells. The recombinant sFGL1 was induced expression by isopropyl-β-d-thiogalactoside (IPTG) and the purified sFGL1 was used as an antigen to produce mouse monoclonal antibody (mAb) and rabbit polyclonal antibody (pAb). After identification, a double-antibody sandwich enzyme-linked immunosorbent assay (DAS-ELISA) for sensitive and specific detection of sFGL1 was developed. Swine FGL1 in samples was captured by anti‐sFGL1 mAb followed by detection with anti‐sFGL1 rabbit pAb and HRP-conjugated goat anti-rabbit IgG. The limit of detection of the developed sFLG1-DAS-ELISA is 35 pg/ml with recombinant sFLG1. Besides, it does not show cross‐reactivity with the control protein. Then serum samples of PRRSV-negative and -positive pigs were tested with the established DAS-ELISA and calculated according to the equation of y=0.0735x+0.0737. The results showed that PRRSV infection enhanced the serum FGL1 levels significantly. Our research provides a platform for the research on the functional roles of swine FGL1.

## Highlights

- Fibrinogen-like protein 1 (FGL1) is a major ligand of lymphocyte activating gene 3 (LAG3) and the FGL1–LAG3 interaction reveals a new immune escape mechanism.
- Our double-antibody sandwich ELISA allows sensitive and specific detection of swine FGL1 in serum samples which can provide technical support for exploring the role of FGL1 in immunosuppressive diseases of pigs.

## Introduction

Fibrinogen-like protein 1 (FGL1), also known as hepatocyte-derived fibrinogen-related protein 1 (HFREP1) or Hepassocin (HPS), is a hepatocyte secreted protein that was initially cloned from liver tissue and has been demonstrated to be over-expressed in human hepatocellular carcinoma (1–3). This protein belongs to the fibrinogen superfamily and it contains a fibrinogen-related domain in its C-terminal portion but lacks three functional domains of platelet binding site, crosslinking region, and thrombin-sensitive site (4, 5). The exact role of FGL1 in the liver is controversial. It has been reported that exogenous FGL1 promotes the proliferation of normal hepatocytes, stimulates hepatocyte proliferation *in vivo*, and prevents the rat liver injury induced by D-galactosamine and carbon tetrachloride (CCl4) (6, 7). Paradoxically, FGL1 has also shown a suppressive effect on the growth of hepatocellular carcinoma cells (8, 9). It has been reported that FGL1 is abundantly associated with the fibrin matrix after clot formation and it may play a role at extrahepatic sites including the regulation of fibrin polymerization (10). FGL1 is present in the plasma of rats and a stable fraction of it remains free in the serum at all times. This unbound fraction may have other biologic roles distinct from that in liver regeneration and clot formation (2). It has been reported that FGL1 confers gefitinib resistance in the NSCLC cell line PC9/GR by regulating the PARP1/caspase 3 pathway. Hence, FGL1 maybe a potential therapeutic target to improve the treatment response of NSCLC patients with acquired resistance to gefitinib (11). Also, It is found that the plasma FGL1 concentrations were significantly higher in the obese group than those in the normal weight group and FGL1 may induce adipogenesis through an ERK1/2-C/EBPβ-dependent pathway in 3T3-L1 adipocytes. So, FGL1 might be a novel therapeutic target to combat obesity (12).

Lymphocyte activation gene 3(LAG3), also known as CD223, is a coinhibitory molecule mainly expressed on activated CD4+ and CD8+ T cells as well as natural killer cells, T regulatory cells (Tregs), and plasmacytoid dendritic cells (DCs). It was found to impede T cell expansion, control the number of memory T cells, suppress Treg activity and T cell homeostasis (13–16). Thus, modulation of the LAG3 pathway has the potential to impact autoimmunity and infections as well as cancer (17). A recent study has demonstrated that FGL1 is a major immune inhibitory ligand for LAG3, and the interaction between FGL1 and LAG3 can inhibit the anti-tumor effect of T cells *in vitro* and *in vivo*, while FGL1 gene silencing can promote the anti-tumor effect of T cells in the mouse model (18), thus reveals a new immune escape mechanism.

Porcine reproductive and respiratory syndrome (PRRS), caused by porcine reproductive and respiratory syndrome virus (PRRSV) infection, commonly known as blue ear disease, is one of the most serious infectious diseases affecting the global pig production. Persistent viral infection of lymphoid tissues is one major characteristic of this disease, and the mechanism has not been fully elucidated (19–21). Studies from humans and mice have indicated that the up-regulation of coinhibitory molecules on host cells is one of the important reasons for the formation of persistent infections, tumors, and autoimmune diseases (22, 23). Does sFGL1 function as a major inhibitory receptor of LAG3 on activated porcine T cells? Whether the interaction between sFGL1 and LAG3 leads to inhibition of the proliferation and activity of activated T cells causing the persistence of a variety of porcine virus infections is unknown at present. Currently, several well-characterized assays to detect FGL1 are available for humans and mice samples; however, sFGL1 has not been well characterized. In this study, the sFGL1 gene was amplified by RT-PCR. Then the purified recombinant protein of sFGL1 was obtained by prokaryotic expression. The mouse mAb and rabbit pAb against sFGL1 were prepared by immunizing mice and rabbits, respectively. Based on the identification of antibodies, a double-antibody sandwich enzyme-linked immunosorbent assay method was established to detect sFGL1. The results of this study will accumulate data and provide a basis for the research of FGL1 in swine immune suppressive diseases.

## Materials and Methods

### Tissue, Vector, Cells, mAb, and Sera

Fresh swine liver tissue was collected from a slaughterhouse in Yangling Agricultural High-tech Industry Demonstration Zone, Shaanxi Province, and brought back to the laboratory at low temperature and stored in −80°C refrigerator for future use. pQE-30 vector, SP2/0 cells, and mAb2-5G2 (24), an anti-idiotypic mAb with IgG1 heavy chain and κ light chain, were preserved in our laboratory. Serum samples of SPF pigs infected with PRRSV JAX1 strain, PRRSV-positive pigs, and PRRSV-negative pigs are validated serum samples kept in our laboratory.

### Experimental Animals

BALB/c mice and New Zealand rabbits were purchased from Chengdu Dossy Experimental Animals Co., LTD.

### Expression and Purification of Swine FGL1

**Scheme 1:**
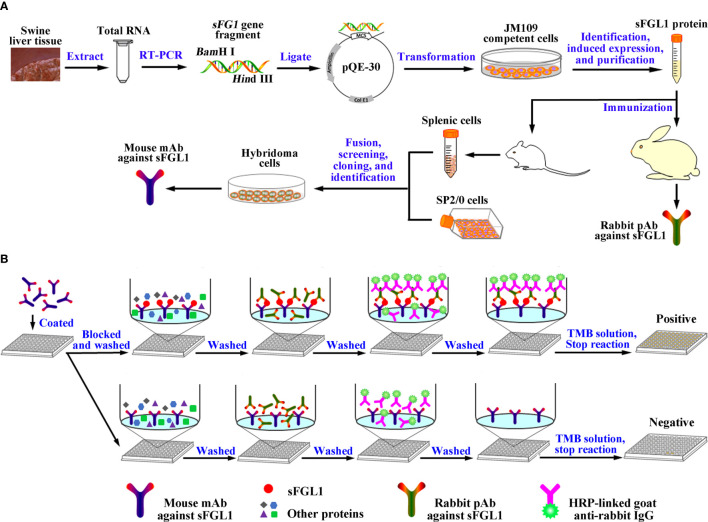
Graphic abstract. **(A)** Diagram for the acquisition of the mouse mAb and rabbit pAb against sFGL1. **(B)** Designation of the developed DAS-ELISA.

Swine FGL1 recombinant protein without signal peptide was expressed and purified (**Scheme 1A**). Briefly, total RNA was extracted from the fresh liver tissue using the RNAiso Plus reagent (TaKaRa, Japan), and cDNA was synthesized using the PrimeScript^®^ RT reagent kit (TaKaRa, Japan). The full-length sFGL1 gene was amplified with primers sFGL1-peF and sFGL1-peR (**Table 1**) using PrimeSTAR^®^ Max DNA polymerase (TaKaRa, Japan) according to the manual instructions. After identification, the truncated sFGL1 gene without sequence encoding signal peptide was amplified with primer pairs sFGL1-peF-dSP and sFGL1-peR (**Table 1**). The PCR product was recovered from 1% agarose gel and inserted into the pQE-30 vector (Qiagen, German). Then, the identified plasmid pQE30-sFGL1 was transformed into *Escherichia coli* (*E. coli*) JM109 competent cells (TaKaRa, Japan). The FGL1 protein expression was induced for 6 h at 37°C by the addition of 1 mmol/L isopropyl-β-d-thiogalactoside (IPTG, TaKaRa, Japan) when the A_600nm_ of cultures reached 0.6 to 0.8. The cultures were centrifuged at 10,000*g* for 10 min at 4°C. After that, the *E. coli* cell pellet obtained from 1 L culture was resuspended in 25 ml PBS and sonicated on ice. After centrifugation at 15,000*g* for 20 min at 4°C, the precipitate was resuspended with solubilization buffer(100 mmol/L Na_2_PO_4_, 10 mmol/L Tris–HCl, 8 mol/L urea, pH 8.0) and the targeted protein was purified with cOmplete™ His-Tag Purification Resin according to the instructions (Roche, Switzerland). The expression and the purification effect were analyzed by sodium dodecyl sulfate-polyacrylamide gel electrophoresis (SDS-PAGE) and Western blot. Eluates containing recombinant sFGL1 were pooled and dialyzed. Dialyzed sample was concentrated using an Amicon Ultra centrifugal concentrator (Millipore, USA) with 8–14 kDa molecular weight cutoff. The protein concentration of the recombinant sFGL1 was determined with a BCA Protein Assay Kit (Thermo Fisher Scientific, USA).

**Table 1:** Primers used in this study.

Primer’s name	Sequence (5’-3’)
**sFGL1-peF**	CGGGATCCATGGGAAAAATGGCAAAGA (*Bam*H I)^a^
**sFGL1-peF-dSP**	CGGGATCCATGCTCGAGGAGGACTGC (*Bam*H I)
**sFGL1-peR**	CCCAAGCTTAACAATATTTGGAATAAAATCATTT 3’ (*Hin*d III)
**sFGL1-eeF-3.1**	CCCAAGCTTATGGGAAAAATGGCAAAGA (*Hin*d III)
**sFGL1-eeR-3.1**	CGGGATCCAACAATATTTGGAATAAAATCATTT (*Bam*H I)

^a^The underlined are BamH I and Hind III restriction enzyme sequences.

### Rabbit Polyclonal Antibody Production

Two New Zealand female rabbits were immunized with purified recombinant sFGL1 followed conventional subcutaneous injection with slight modifications (25) (**Scheme 1A**). Routinely, purified recombinant sFGL1 (1 mg/ml) was emulsified with an equal amount of Freund’s complete adjuvant (Sigma‐Aldrich, USA) (v/v) for primary immunization (1 mg/rabbit). The protein was emulsified with an equal amount of Freund’s incomplete adjuvant (Sigma‐Aldrich, USA) (v/v) for the two subsequent booster immunization at two-week intervals (0.5 mg/rabbit). Seven days after the third immunization, sera were collected, repackaged, and stored at −20 °C. The titers of pAb were determined with an indirect enzyme-linked immunosorbent assay (I-ELISA), using the recombinant sFGL1 protein (400 ng/well) as coating antigen.

### Mouse Monoclonal Antibody Production

To produce hybridomas cells secreting anti‐sFGL1‐specific mAbs (**Scheme 1A**), purified recombinant sFGL1 (50 μg/mouse) was emulsified with Freund’s complete adjuvant for primary immunization. Freund’s incomplete adjuvant was used in the next three immunization with 2-week intervals. The antibody titers of mice were detected with an I-ELISA after the 4th immunization. The mouse with the highest antibody titer received the final booster dose intraperitoneally 3 days before fusion. Splenocytes of the mouse were fused with SP2/0 cells. RPMI-1640 medium containing HAT/HT (Sigma‐Aldrich, USA) and 10% FBS (Gibco, USA) was used for screening (26). Subsequently, the positive clones determined with an I-ELISA by detecting the supernatant of hybridoma cells were expanded culture. The isotype of mAbs was determined with an IsoStrip™ Mouse Monoclonal Antibody Isotyping Kit (Roche, Switzerland). After preliminary characterization, the positive hybridoma cells (5×10^5^ cells/mouse) were injected into paraffin oil sensitized mice for mAb production in the form of ascites. Antibody titers of the ascites were detected with an I-ELISA and the mAbs were purified by precipitation with 50% (v/v) saturated ammonium sulfate followed by affinity chromatography with Protein G Resin (Genscript, China). The concentration of mAbs was determined using a BCA Protein Assay Kit.

### Construction of pcDNA3.1/V5-HisB-sFGL1 Eukaryotic Expression Plasmid

The full-length sFGL1 gene was amplified using primers sFGL1-eeF-3.1 and sFGL1-eeR-3.1 (**Table 1**). The PCR product was recovered from 1% agarose gel and inserted into the pcDNA3.1/V5-HisB vector (Invitrogen, USA) and the recombinant plasmid pcDNA3.1/V5-HisB-sFGL1 was confirmed by double digestion and sequencing.

### Western Blot Analysis

HEK 293T cells transfected with pcDNA3.1/V5-HisB-sFGL1 plasmid were collected at 48 h after transfection. After washing with PBS, cell samples were lysed with NP40 lysis buffer (Beyotime, China) and the concentration of the samples was determined using a BCA Protein Assay Kit. Equal amounts of protein were loaded and subjected to SDS-PAGE and then transferred to PVDF membranes (Millipore, USA) using a BIO-RAD Mini Trans-Blot Electrophoretic Transfer Cell. The membranes were blocked with 5% skim milk (BioFroxx, Germany) and then incubated with indicated primary antibodies overnight at 4°C, followed by HRP-linked secondary antibodies. Alpha-tubulin or GAPDH were used as a loading control. The protein bands were visualized using ChemiDoc™ MP Imaging System (Bio-Rad, USA). The experiment was repeated three times.

### Indirect Immunofluorescence Assay (IFA)

HEK 293T Cells transfected with pcDNA3.1/V5-HisB-sFGL1 plasmid were fixed with 4% paraformaldehyde for 10 min at RT, washed with PBS, permeabilized with PBS containing 0.25% Triton X-100 for 10 min at RT, then washed and blocked with PBS containing 1% BSA. After washing, the cells were incubated with primary antibodies for 1 h at 37°C, washed with PBS, and incubated with the corresponding secondary antibody. Finally, the slides were stained with DAPI and visualized using Leica microsystems (Leica AF6000, Germany). The experiment was repeated three times.

### Monoclonal Antibody Epitope Analysis

For monoclonal antibody epitope analysis, an ELISA-mediated antibody overlap experiment was performed (27, 28) and the experiment was repeated three times. A mAb, mAb2-5G2 with IgG1 heavy chain and κ light chain, was used as an isotype control. An additive index (AI), which compared the optical densities (ODs) obtained for the two mAbs assayed under standardized conditions (either alone or in a mix) was calculated for each pair of mAbs using the following formula,

$$\text{AI}=(((2\times {\text{A}}_{1+2}/(\text{A}1+\text{A}2))-1)\times 100,$$

where A1 and A2 refer to the ODs obtained when the mAbs were assayed separately, and A_1 + 2_ refers to the OD obtained when the same amounts of the two mAbs were pooled in the same well. If both mAbs bound to the same epitope, the AI would be negligible; otherwise, the AI would be near 100 if the two epitopes were topographically unrelated.

### Establishment and Optimization of a DAS-ELISA

Using prepared mouse mAb against sFGL1 as capture antibody and prepared rabbit pAb as detection antibody, a DAS-ELISA method for sFGL1 sensitive detection was established (**Scheme 1B**). To achieve optimal DAS-ELISA performance, various experimental conditions were optimized and all experiments were repeated three times.

Firstly, the optimal coating concentration of the mAb (0.5, 1, 2, 4, and 8 μg/well) and the optimal dilution of the pAb (1:100, 1:500, 1:1,000, 1:2,000, 1:4,000) were determined using checkerboard titration. The optimal conditions were determined when the largest ratio of OD_450nm_ values between the positive samples and negative control samples (P/N) were obtained.

Secondly, using the determined optimal coating concentration of mAb and the optimal dilution of pAb, the incubation time for samples was set to 30, 45, 60, 75, and 90 min for DAS-ELISA.

Thirdly, using the determined optimal coating concentration of mAb, the optimal dilution of pAb, and the determined incubation time for samples, the incubation times for the detection antibody (30, 45, and 60 min), the HRP-linked antibody (30, 45, and 60 min), and the colorimetric reaction (10, 15, and 20 min) were optimized in turn.

### Preparation Standard Curves

Following the determined procedure, 2,000, 1,000, 500, 250, 125, 62.5, 31.25, 15.625, 7.8125, 3.90625, 1.953125, 0.9765, and 0 ng/ml purified recombinant sFGL1 was used as antigen to perform the DAS-ELISA and the experiment was repeated three times. The standard curves of sFGL1 detection by the DAS-ELISA were drawn with sFGL1 concentration as the horizontal coordinate and OD_450nm_ values as the vertical coordinate.

The limit of detection (LOD) was calculated by the following standard formula.

$${\text{OD}}_{\text{LOD}}=\text{Mean}({\text{OD}}_{\text{blank}})+3({\text{SD}}_{\text{blank}})$$

where OD_LOD_ is the optical density corresponding to the LOD, OD_Blank_ is the optical density of the blank, and SD_blank_ is the standard deviation of the blank from all repeats (26, 29, 30).

### Applicability of the Developed DAS-ELISA

Using the developed DAS-ELISA method, mAb2-5G2 (24), a mAb with IgG1 heavy chain and κ light chain, used as a control capture antibody, twenty PRRSV-positive pig sera and four PRRSV-negative pig sera were detected. Then serum samples of SPF pigs infected with PRRSV JAX1 strain, 81 PRRSV-negative pig, and 86 PRRSV-positive pig were diluted and detected with the developed DAS-ELISA. All samples were tested with duplicate wells and the FGL1 contents were calculated according to the standard curve.

### Statistical Analysis

Statistical analysis and inferences were performed using GraphPad Prism software 8.4 (GraphPad Software, USA) using a one-way analysis of variance(one-way ANOVA) followed by Tukey post-test: compare all pairs of columns. Comparisons between groups were considered statistically significant at p <0.05.

## Results

### Amplification of sFGL1 Gene and Expression of sFGL1 Protein

**Figure 1:**
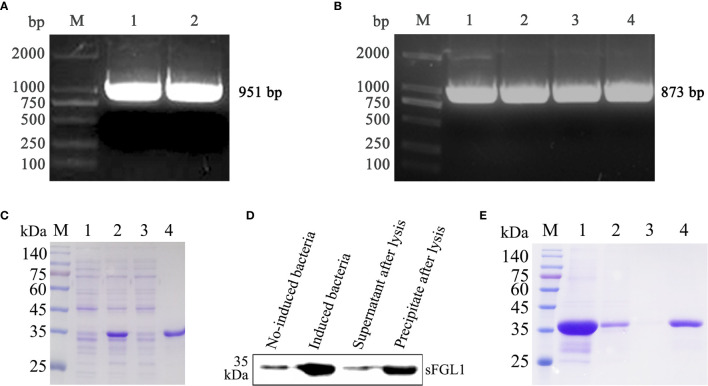
Expression, identification, and purification of recombinant sFGL1. **(A)** Amplify full-length sFGL1 gene using RT-PCR method. M, DL2000 DNA marker; 1–2, Full-length sFGL1 gene. **(B)** Amplify sFGL1 gene without sequence encoding the signal peptide. M, DL2000 DNA marker; 1–4, sFGL1 gene without fragment encoding the signal peptide. **(C)** Identify sFGL1 expression by SDS-PAGE and Coomassie brilliant blue staining. M, Prestained protein marker(SMOBIO PM2500); 1, No-induced JM109-pQE30-sFGL1; 2, Induced JM109-pQE30-sFGL1; 3, Supernatant after lysis; 4, Precipitate after lysis. **(D)** Identify sFGL1 expression by Western blot. **(E)** Purification of sFGL1. M, Prestained protein marker(SMOBIO PM2500); 1, Sample loaded; 2, Effluent; 3, 5 mmol/L imidazole eluent; 4, Purified sFGL1.

The total RNA of fresh swine liver tissue was extracted and the full-length sFGL1 gene was amplified using the RT-PCR method. Sequence analysis showed that the sFGL1 gene is 951 bp in length (**Figure 1A**). The nucleotide sequence is completely consistent with the CDS (120–1,030) of the predicted *Sus scrofa* FGL1 sequence on NCBI (NCBI reference sequence: XM_021077953.1), and the derived amino acid sequence was completely consistent with the logged *S. scrofa* FGL1 (NCBI reference sequence: XP_020933612.1). A, G, T, and C were 301, 237, 242, and 171, respectively, with A + T accounting for 57.10% and G + C for 42.90%. The nucleotide sequence was submitted to the GenBank database of NCBI, and the GenBank sequence number is MK813968. The sFGL1 gene without signal peptide sequences is 873 bp (**Figure 1B**). This fragment was ligated in pQE-30 vector and the identified plasmid pQE30-sFGL1 was transformed into *E. coli* JM109 competent cells. After induced with 1 mmol/L IPTG at 37 °C for 6 h, recombinant sFGL1 protein was about 35 kDa and mainly expressed in the form of inclusion bodies (**Figure 1C**). When detected by Western blot with *ProteinFind* ^®^ Anti-His Mouse Monoclonal Antibody (TransGen Biotech, China) as the primary antibody, the imprinted strip appeared at the expected location (**Figure 1D**). The purified recombinant sFGL1 protein was obtained by dialysis and concentration after His-Tag Resin purification (**Figure 1E**).

### Characteristics of Rabbit pAb Against sFGL1

**Figure 2:**
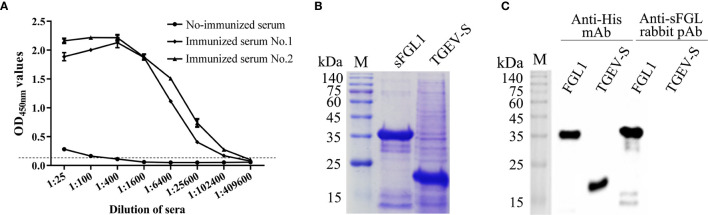
Characteristics of rabbit pAb against sFGL1. **(A)** Titers of antibody against sFGL1 in the sera of immunized rabbits. **(B)** Identify expressed sFGL1 and TGEV-S protein by SDS-PAGE and Coomassie brilliant blue staining. The TGEV-S protein was used as a control protein. **(C)** Identify rabbit pAb against sFGL1 by Western blot.

After immunized with the purified recombinant sFGL1, the serum antibody titers of the rabbits were detected with an I-ELISA. The results showed the antibody titers of the two rabbits all reached 1:102,400 after three immunizations (**Figure 2A**). The expressed sFGL1 and control protein (pQE30-TGEV-S protein) were used to identify the specificity of the prepared pAb by Western blot. The results indicated that the pAb only reacted specifically with sFGL1 and did not react with the control protein (**Figures 2B, C**).

### Characteristics of Mouse mAb Against sFGL1

**Figure 3:**
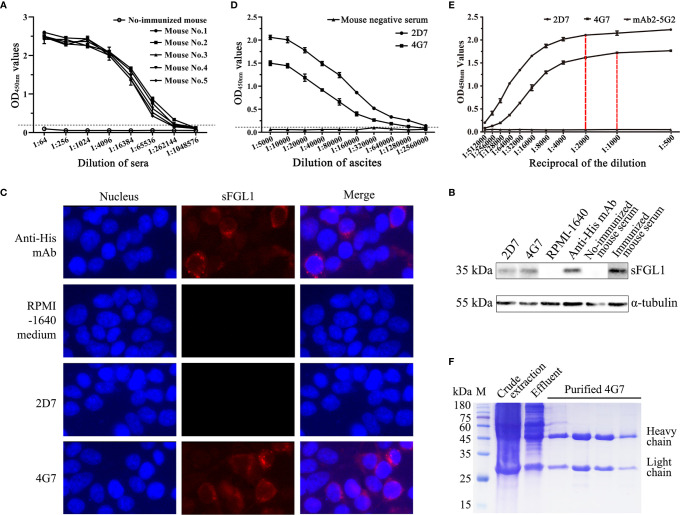
Characteristics of mouse mAb against sFGL1. **(A)** Titers of antibody against sFGL1 in the sera of immunized mice. **(B)** Identify the reaction of 2D7 and 4G7 with sFGL1 expressed by HEK 293T cells with the Western blot method. **(C)** Identify the reaction of 2D7 and 4G7 with sFGL1 expressed by HEK 293T cells with IFA. **(D)** Titers of 2D7 and 4G7 in ascites. **(E)** The saturation curves of 2G7 and 4G7 with sFGL1. The plates were coated with 50 ng purified recombinant sFGL1 per well, different diluted 2D7 and 4G7 were used to perform an I-ELISA, mAb2-5G2 purified from ascites was used as a negative control. **(F)** Identify purified 4G7 by SDS-PAGE and Coomassie brilliant blue staining.

After immunized four times with the purified recombinant sFGL1, the serum antibody titers of five mice were all over 1:262,144 detected with an I-ELISA method (**Figure 3A**). After fusion and screening with RPMI-1640 medium containing HAT or HT, two hybridoma cell lines that can stably secrete antibodies against sFGL1 were finally obtained, named 2D7 and 4G7, respectively. According to the test results of the IsoStrip™ Mouse Monoclonal Antibody Isotyping Kit, both heavy chain and light chain of mAb 2D7 and 4G7 are IgG1 and κ, respectively (**Figure S1**). HEK 293T cells transfected with pcDNA3.1/V5-HisB-sFGL1 for 48 h were collected and lysed. 2D7 and 4G7 were used as primary antibodies for Western blot analysis, respectively. Meanwhile, Anti-His Mouse Monoclonal Antibody and sFLG1 positive serum of mice were used as positive control, and RPMI-1640 medium was used as negative control. The results showed that both 2D7 and 4G7 could react with sFGL1 expressed by HEK 293 T cells (**Figure 3B**). Indirect immunofluorescence assay was carried out on HEK 293 T cells transfected with pcDNA3.1/V5-HisB-sFGL1 plasmid using 2D7 and 4G7 mAbs as primary antibodies. The results showed that 4G7 could react with sFGL1 expressed by HEK 293 T cells, while 2D7 could not react with sFGL1 expressed by HEK 293 T cells (**Figure 3C**).

The two hybridoma cells were intraperitoneally injected into mice pretreated with liquid paraffin to collect ascites. The antibody titers of 2D7 and 4G7 in ascites were 1:2,560,000 and 1:1,280,000, respectively (**Figure 3D**). To analyze the monoclonal antibody epitope, the saturation curves of the antigen by each antibody should be determined firstly (27). The results were shown in **Figure 3E**. It can be seen that for the two mAbs tested, a plateau is practically reached for 1:2,000 (2D7) and 1:1,000 (4G7) dilutions, respectively. Then the ELISA additive test was performed with the determined dilutions and the additive index (AI) was 27.2%, which means the additive effect of 2D7 and 4G7 was not obvious (**Table 2**). After coarse extract by saturated ammonium sulfate, the prepared ascites were purified with affinity chromatography with Protein G, and SDS-PAGE results showed that purified 4G7 were obtained (**Figure 3F**).

**Table 2:** Results of ELISA additivity test.

Antibody	OD_450 nm_	AI	Possible match
**2D7 (1:2,000)**	1.802 ± 0.020	–	–
**4G7 (1:1,000)**	1.343 ± 0.006	–	–
**2D7 (1:2,000) + 4G7 (1:1,000)**	2.001 ± 0.034	27.2%	<50%

### Establishment and Optimization of the DAS-ELISA

The optimal coating amount of capture antibody and the dilution of detection antibody were determined by checkerboard titration assay. The results showed that the optimal amount of 4G7 was 1 μg/well, and the optimal dilution of rabbit pAb was 1:1,000 (**Table 3**).

**Table 3:** Determine the optimal concentration of capture antibody and dilution of detection antibody by orthogonal assay.

Amounts of capture antibody (μg/well)	Dilution of detection antibody									
	1:100		1:500		1:*1,000* ^a^		1:2,000		1:4,000	
	P	N	P	N	P	N	P	N	P	N
**0.5**	2.644 ± 0.052	0.221 ± 0.000	2.517 ± 0.028	0.116 ± 0.019	2.425 ± 0.040	0.114 ± 0.008	2.338 ± 0.008	0.102 ± 0.003	1.821 ± 0.006	0.092 ± 0.002
**P/N**	11.964		21.698		21.272		22.922		19.793	
***1***	2.651 ± 0.033	0.118 ± 0.011	2.565 ± 0.101	0.07 ± 0.001	2.485 ± 0.049	0.063 ± 0.004	2.344 ± 0.04	0.063 ± 0.007	2.123 ± 0.076	0.060 ± 0.004
**P/N**	22.466		36.643		39.444		37.206		35.383	
**2**	2.748 ± 0.018	0.116 ± 0.028	2.715 ± 0.02	0.071 ± 0.009	2.611 ± 0.051	0.067 ± 0.001	2.412 ± 0.071	0.062 ± 0.016	2.175 ± 0.087	0.059 ± 0.000
**P/N**	23.690		38.239		38.970		38.903		36.864	
**4**	2.720 ± 0.003	0.127 ± 0.006	2.721 ± 0.031	0.080 ± 0.000	2.647 ± 0.002	0.068 ± 0.004	2.443 ± 0.04	0.063 ± 0.001	2.223 ± 0.001	0.058 ± 0.001
**P/N**	21.417		34.013		38.926		38.778		38.328	
**8**	2.721 ± 0.028	0.143 ± 0.023	2.703 ± 0.009	0.075 ± 0.007	2.556 ± 0.000	0.069 ± 0.008	2.433 ± 0.016	0.064 ± 0.011	2.182 ± 0.062	0.059 ± 0.001
**P/N**	19.028		36.040		37.043		38.016		36.983	

^a^The italics represent the selected amount of capture antibody and the dilution of detection antibody.

**Figure 4:**
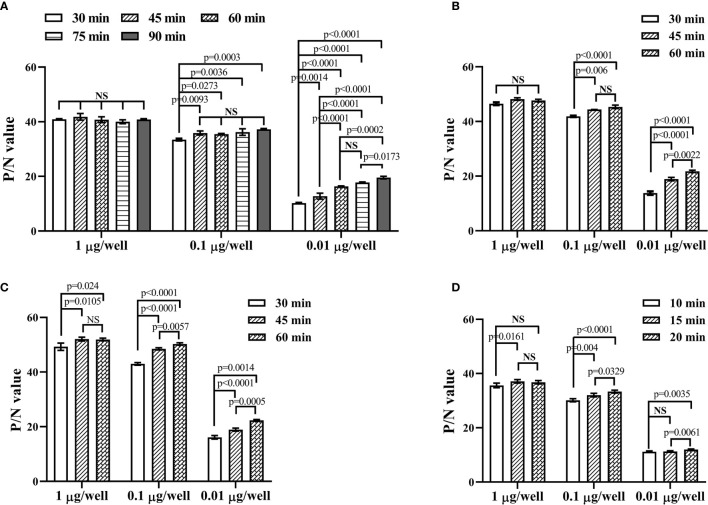
Optimize the reacting conditions of the developed DAS-ELISA. **(A)** Determination of the incubation time for samples. **(B)** Determination of the incubation time for the detection antibody. **(C)** Determination of the incubation time for the HRP-linked goat anti-rabbit IgG. **(D)** Determination of the colorimetric reaction time. NS, nonsignificance.

Using the determined concentration and dilution, 1, 0.1, and 0.01 μg purified recombinant sFGL1 were added to each well and incubated at 37 °C for 30, 45, 60, 75, or 90 min respectively for DAS-ELISA detection. The results showed, when the amount of sFGL1 was 1 μg/well, with incubation 30, 45, 60, 75, or 90 min respectively, the P/N values changed from 40.016 to 41.836, which means an excess of antigen. With the prolongation of antigen incubation time, the P/N values increased slightly all the time when the amount of sFGL1 was 0.1 μg/well. However, when the antigen dosage was 0.01 μg/well, the P/N value increased significantly when incubated for 60 min (**Table 4** and **Figure 4A**). Combined with the requirements of actual detection, the sample incubation time was set to 60 min.

**Table 4:** Determination of the optimal incubation time for samples.

Con. of Antigen (μg/well)		Incubation time for antigen (min)				
		30	45	60	75	90
**sFGL1**	**1**	2.537 ± 0.05	2.552 ± 0.074	2.527 ± 0.066	2.481 ± 0.045	2.655 ± 0.017
	**P/N**	40.919	41.836	40.758	40.016	40.846
	**0.1**	2.079 ± 0.022	2.188 ± 0.045	2.2 ± 0.016	2.246 ± 0.073	2.421 ± 0.019
	**P/N**	33.532	35.869	35.484	36.226	37.246
	**0.01**	0.634 ± 0.005	0.78 ± 0.067	1.015 ± 0.008	1.103 ± 0.013	1.272 ± 0.031
	**P/N**	10.226	12.787	16.371	17.790	19.569
**Negative control**		0.062 ± 0.001	0.061 ± 0.003	0.062 ± 0.000	0.062 ± 0.002	0.065 ± 0.005

Then the incubation times for the detection antibody (30, 45, and 60 min), the HRP-linked goat anti-rabbit IgG (30, 45, and 60 min), and the substrate (10, 15, and 20 min) were determined in turn. To detect lower levels of sFGL1 in samples, they were set to 60, 60, and 20 min, respectively (**Figures 4B–D**).

After the conditions were optimized, the procedure of the developed DAS-ELISA was performed as follows.

(1) Ninety-six-well plates (Corning-Costar, USA) were coated with 100 μl mouse anti-sFGL1 mAb 4G7 (10 μg/ml in 0.01 mol/L PBS, pH 7.2) as the capture antibody overnight at 4 °C;

(2) After washing three times(2 min/time) with PBS containing 0.05% Tween-20(PSB’T, V/V), the plates were blocked with 200 μl blocking buffer (PBS’T containing 5% skim milk, SM-PSB’T, W/V) at 37°C for 1 h;

(3) After wash, 100 μl sample (diluted 1:20 with PBS’T) was added to the wells and incubated 60 min at 37°C. Purified recombinant sFGL1 was used as a positive control;

(4) After wash, 100 μl rabbit anti-sFGL1 pAb diluted 1:1,000 was added to each well and incubated 60 min at 37°C;

(5) After wash, 100 μl peroxidase-conjugated AffiniPure Goat Anti-Rabbit IgG (H + L) (Jackson ImmunoResearch, USA) diluted 1:3,000 was added to each well and then incubated 60 min at 37°C;

(6) After wash, 100 μl 3,3′,5,5′-Tetramethylbenzidine (TMB, Sigma-Aldrich, USA) substrate was added to each well and then incubated 20 min at RT in the dark;

(7) The colorimetric reaction was terminated by adding 50 μl 3 mol/L H_2_SO_4_ to every well. The OD_450nm_ values were then read using an automated ELISA plate reader (Bio-Rad, USA) (**Scheme 1B**).

### The Standard Curve

**Figure 5:**
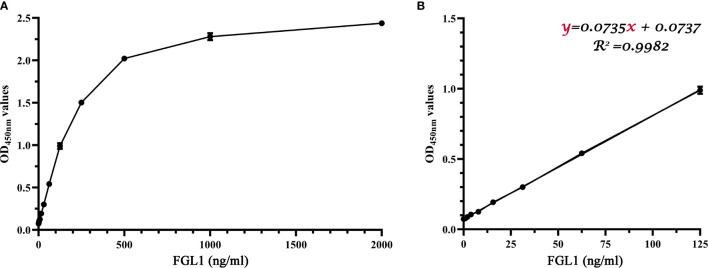
Standard curves of the developed DAS-ELISA. **(A)** A parabolic standard curve of the DAS-ELISA. **(B)** A linear detection line from ‘A’ linear regression shows a working range from 0 to 125 ng/ml (R^2^ = 0.9982).

Following the determined procedure, 2,000, 1,000, 500, 250, 125, 62.5, 31.25, 15.625, 7.8125, 3.90625, 1.953125, 0.9765, and 0 ng/ml purified recombinant sFGL1 was used as antigen to perform the DAS-ELISA. The standard curves for sFGL1 detection by the DAS-ELISA were drawn with sFGL1 concentrations as the horizontal coordinate and OD_450nm_ values as the vertical coordinate (**Figure 5**). The results showed that from 0 to 125 ng/ml, there was a good linear relationship between antigen concentrations and OD_450nm_ values (y = 0.0735x + 0.0737, R^2^ = 0.9982) (**Figure 5B**). Blank control was repeated in 20 individual experiments resulting in an average OD_450nm_ value of 0.0735 with a standard deviation of 0.0009. So the limit of detection was determined 35 pg/ml.

### Applicability of the Developed DAS-ELISA

**Figure 6:**
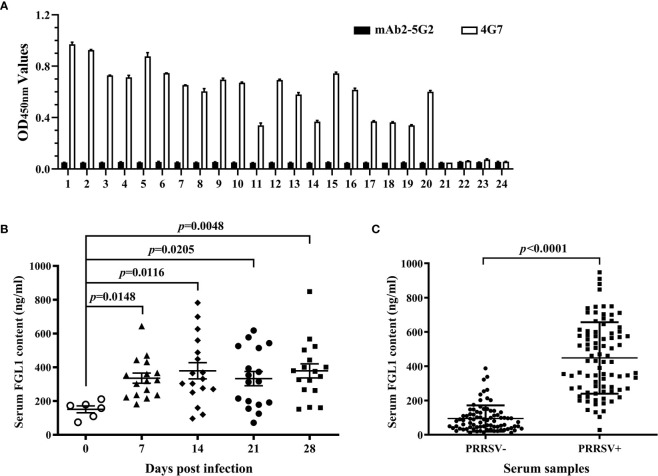
Applicability of the developed DAS-ELISA. **(A)** Twenty PRRSV-positive pig sera and four PRRSV-negative pig sera were detected with the developed DAS-ELISA. mAb2-5G2 was used as a control capture antibody. **(B)** Series serum samples of SPF pig infected with PRRSV JAX1 strain were analyzed with the developed DAS-ELISA. **(C)** Clinically PRRSV-negative and -positive pig serum samples were analyzed with the developed DAS-ELISA.

Using the developed DAS-ELISA, twenty PRRSV-positive pig sera and four PRRSV-negative pig sera were detected. An anti-idiotypic mAb (mAb2-5G2) (24) with the same class of heavy chain and the same type of light chain as 4G7 was used as a control capture antibody (**Figure 6A**). As shown in **Figure 6A**, 4G7 can capture sFGL1 in sera, while mAb2-5G2 did not. Then series serum samples of SPF pigs infected with PRRSV JAX1 strain were detected and the FGL1 contents were calculated according to the equation of y=0.0735x+0.0737. The results showed that FGL1 contents in sera increased significantly at different days after PRRSV infection(*P <*0.05) (**Figure 6B**). Results of 81 clinically PRRSV-negative pig sera and 86 clinically PRRSV-positive pig sera also showed that PRRSV infection led to a significant increase in serum FGL1 levels (*P <*0.0001) (**Figure 6C**).

## Discussion

FGL1, a member of the fibrinogen family, is a liver-secreted protein. It can promote hepatocyte proliferation, protect against liver injury, and act as a tumor suppressor in hepatocellular cancer (2, 6, 31). Recently, it was reported that FGL1 is a major ligand of LAG3, and genetic ablation or mAbs blocking the FGL1-LAG3 interaction enhanced T cell responses and promoted anti-tumor immunity (18). Human and rodent FGL1 can be detected in culture media of expressing cells and in the plasma of humans and rodents (32, 33). Currently, several well-characterized assays to detect FGL1 are available for humans and mice samples, however, sFGL1 has not been well characterized. Double antibody sandwich ELISA (DAS-ELISA) has high sensitivity and specificity and can accurately quantify antigens with easy experimental operation. In the present study, two specific mouse mAbs and rabbit pAb against recombinant swine FGL1 were prepared and an optimized DAS-ELISA method was developed for swine FGL1 detection at protein level, which used the mouse mAb 4G7 and rabbit pAb against swine FGL1 as the capture and detection antibodies, respectively. In this method, parabolic and linear standard curves were both generated with the valid detection scopes of 0–2,000 and 0–125 ng/ml, respectively. The specificity of this method was also proved to be well by using mAb2-5G2, a mAb with the same class of heavy chain and the same type of light chain as 4G7, as a control capture antibody. The results suggested that the developed DAS-ELISA could be a convenient and simple assay to quantify swine FGL1.

*Escherichia coli* (*E. coli*) expression system is the earliest developed and most widely used classical expression system in gene expression technology, which has been widely used in scientific research and industrial users to express various recombinant proteins. Compared with other expression systems, it has the characteristics of high expression level of the target gene, short culture cycle, strong anti-pollution ability, and relatively low cost (34). In this study, sFGL1 without signal peptide was expressed by the *E. coli* expression system. After identification and purification, pAbs were prepared by immunizing New Zealand rabbits and mAbs were prepared by immunizing BALB/c mice and cell fusion technique. Finally, pAbs with titers of 1:102,400 (**Figure 2**) and two mAbs, 2D7 and 4G7 (**Figure 3**), were obtained.

In Western blot identification of recombinant sFLG1, there were also weak imprinted bands in the lane of the no-induced bacteria sample (**Figure 1D**), indicating that the target protein was weakly expressed in the background of the no-induced bacteria, but this did not affect the acquisition of purified recombinant sFGL1 (**Figure 1E**).

The characters of antibodies used in the DAS-ELISA would directly influence the specificity and sensitivity of the detection (35). In this study, to determine the specificity of the obtained rabbit anti-sFLG1 sera, the recombinant TGEV-S protein expressed using the same expression vector and expression system was used as a negative control for Western blot detection. Both sFGL1 and TGEV-S could be detected when anti-His mAb was used as the primary antibody, but when rabbit anti-sFGL1 serum was used as the primary antibody, only sFGL1 could react with the serum to produce an imprinted band (**Figure 2C**), indicating that rabbit pAb is specific and can be used for the establishment of a DAS-ELISA method.

For the mAbs, after identified by Western blot, IFA, and ELISA-mediated antibody overlap experiment, mAb 4G7 was chosen to develop the DAS-ELISA for it can be used in Western blot and IFA analysis (**Figure 3**), although the additive effect of 2D7 and 4G7 was not obvious (**Table 2**). Monoclonal antibodies, with the advantages of strong specificity and high affinity, only recognizing a single antigen determinant, are the best choice of the coated antibody (36–38). We found that mAb 4G7 as the capture antibody exhibited the highest specificity to FGL1 in pig serum samples (**Figure 6**). The ability of pAbs to bind to multiple antigenic epitopes can reduce the missed detection rate and increase the sensitivity of the assay (36). By using mAb 4G7 as capture antibody and rabbit anti-sFGL1 pAb as detection antibody, the limit of detection of the developed DAS-ELISA for sFLG1 was 35 pg/ml with recombinant sFLG1.

At present, the immune function of FGL1 is still unclear. It expresses specifically in normal human liver tissue, but reduced or undetectable in most hepatocellular carcinomas(HCC) specimens at both RNA and protein levels. Furthermore, the reduction or nonexpression of FGL1 is significantly associated with the degree of tumor differentiation (8). Lymphocyte activation gene 3, a type I transmembrane protein, mainly expresses on the activated T lymphocytes, NK cells, and dendritic cells surface. It has a similar regulatory function to PD-1 on T cells, mainly acting as a receptor to deliver inhibitory signals (39). Most literature has reported that MHC class II molecules are the main ligand used by cancer cells to bind LAG3 and thus reduce T cell activity (40–42). Wang et al. reported FGL1 is a major immune inhibitory ligand of LAG3 independent of MHC class II molecules, and the interaction between FGL1 and LAG3 can inhibit the anti-tumor effect of T cells *in vivo* and *in vitro*, thus revealing a new immune escape machine (18). Studies from humans and mice have shown that the up-regulation of co-inhibitory molecules on host cells is one of the important causes of persistent infections, tumors, and autoimmune diseases (22, 23). Programmed death-1 (PD-1)/programmed cell death ligand-1 (PD-L1) and cytotoxic T-lymphocyte-associated protein 4 (CTLA-4) are the coinhibitory molecules that have been studied earlier and deeply. There are already FDA-approved blockers targeting them in human medicine for the treatment of cancer (43). Porcine reproductive and respiratory syndrome (PRRS), caused by porcine reproductive and respiratory syndrome virus infection (PRRSV) and commonly known as blue ear disease, is one of the most serious infectious diseases impacting the global swine industry production. Persistent viral infection of lymphoid tissues is one major characteristic of PRRS, and the mechanism has not been fully elucidated (44–46). The porcine monocyte-derived dendritic cells (MoDC) infected with 1 multiplicity of infection (MOI) PRRSV showed a strain-dependent increase in PD-L1 expression (47). When porcine peripheral blood mononuclear cells (PBMCs) were cultured in the presence of the R98-strain of PRRSV (5 × 10^4^ TCID_50_/ml), the mRNA and protein expression of cytotoxic T-lymphocyte antigen 4 (CTLA-4) increased significantly compared with the control group (48). Those suggested that the pathogenesis of PRRSV is related to the increased expression of PD-L1 and CTLA-4. Our previous study indicated that LAG3 transcription was significantly increased in the lymphoid tissues of PRRSV-infected pigs (data not published). In the present study, FGL1 contents in the samples of 20 PRRSV-positive pig sera and 4 PRRSV-negative pig sera were analyzed with the developed DAS-ELISA. An anti-idiotypic mAb, mAb2-5G2 (24), was used as a control capture antibody. The results showed that 4G7 can capture sFGL1 in sera, while mAb2-5G2 cannot. Moreover, FGL1 levels increased significantly in series serum samples of SPF pigs infected with PRRSV JAX1 strain and serum samples of 86 clinically PRRSV-positive pigs (**Figures 6B, C**). Therefore, based on our results and the above research, we speculate that PRRSV infection raises sFGL1 expression, activates FGL1-LAG3 pathways, and inhibits proliferation and activity of T cells, which results in virus removal decrease and persistent infection. The details need to be further studied.

Taken together, we prepare specific mAbs and pAb against swine FGL1 and develop a double-antibody sandwich ELISA for sensitive and specific detection of swine FGL1 using mAb 4G7 as capture antibodies and rabbit-anti sFGL1 pAb as detection antibody. This method can be used to detected FGL1 levels in serum samples which can provide technical support for exploring the role of FGL1 in immunosuppressive diseases in pigs.

## Data Availability Statement

The datasets presented in this study can be found in online repositories. The names of the repository/repositories and accession number(s) can be found below: https://www.ncbi.nlm.nih.gov/MK813968.

## Ethics Statement

The animal study was reviewed and approved by Committee on Ethical Use of Animals of Northwest A&F University.

## Author Contributions

YM designed the experiments. YM and XZha drew the scheme and figures. XZha performed the experiments. HZ, XZhe, and YJ contributed to the reagents, materials, and analysis tools. YM and XZha analyzed the data. YM, XZha, and E-MZ wrote the paper. All authors contributed to the article and approved the submitted version.

## Funding

This study was supported by grants from the National Natural Science Foundation of China(grant no. 31972675) and the National Training Program of Innovation and Entrepreneurship for Undergraduates (grant no. S202010712070).

## Conflict of Interest

The authors declare that the research was conducted in the absence of any commercial or financial relationships that could be construed as a potential conflict of interest.
