# Supplementary figures and images for: A Double-Antibody Sandwich ELISA for Sensitive and Specific Detection of Swine Fibrinogen-Like Protein 1
Source: Front Immunol. 2021 Apr 23;12:670626. doi: 10.3389/fimmu.2021.670626 (PMC8102871; doi:10.3389/fimmu.2021.670626)

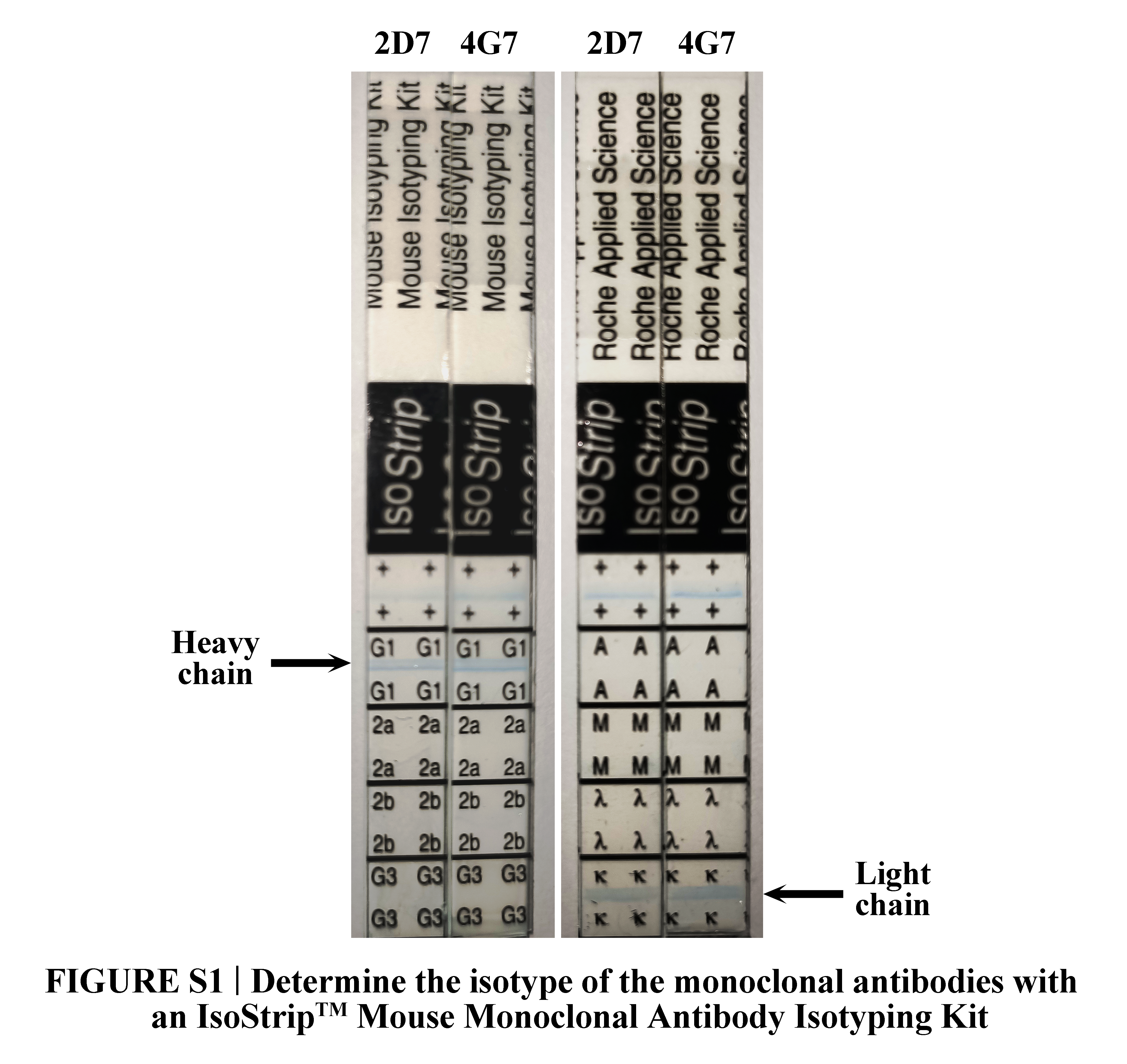

Supplement: Supplementary file 1 [file Image_1.tif]
